# High frequency of HLA class I antigen processing machinery (APM) component upregulation in primary hepatocellular carcinoma tumors

**DOI:** 10.1186/2051-1426-1-S1-P264

**Published:** 2013-11-07

**Authors:** Fabio Grizzi, Barbara Franceschini, Sonia Di Biccari, Paola Bossi, Maurizio Chiriva-Internati, Soldano Ferrone

**Affiliations:** 1 Laboratory of Molecular Gastroenterology, Humanitas Clinical and Research Center, Rozzano, Italy; 2 Laboratory of Quantitative Medicine, Humanitas Clinical and Research Center, Rozzano, Italy; 3 Department of Pathology, Humanitas Clinical and Research Center, Rozzano, Italy; 4 Department of Internal Medicine, Texas Tech University Health Science Center Medical School, Lubbock, TX, USA; 5 Department of Surgery, Massachusetts General Hospital, Harvard Medical School, Boston, MA, USA

## 

Malignant transformation of cells is associated with downregulation of HLA class I APM components in most of the tumors. These defects are clinically relevant, since they are frequently associated with the clinical course of the disease. Only in a few tumors malignancy is associated with the upregulation of HLA class I antigens. Among them is hepatocellular carcinoma (HCC). The frequency of HLA class I APM component upregulation and its clinical significance in HCC are not known. These topics were investigated in the present study, since the resulting information may contribute to assess the therapeutic efficacy of T cell-based immunotherapy for the treatment of HCC. Twenty-one surgically resected primary HCC tumors and autologous adjacent non-malignant tissues were stained with a unique panel of monoclonal antibodies which recognize HLA class I APM components. The staining patterns of the malignant tumors were compared to those of the autologous non malignant tissues. To assess the functional significance of changes in HLA class I APM component expression in HCC tumors, the results of immunohistochemical staining were correlated with the extent of CD8+ cytotoxic T-lymphocyte infiltrate, quantified with a computer-aided image analysis system. In all the HCC tumors, malignant hepatocytes expressed high levels of HLA class I APM components. In contrast these molecules were not detected in normal hepatocytes, although they displayed a low expression in some apparently normal hepatocytes adjacent to the HCC tumor. The HLA class I APM component upregulation in HCC was associated with the extent of CD8+ T cell infiltrate, although this association did not reach the level of statistical significance. Our results corroborate the information in the literature about the lack of HLA class I antigen expression in hepatocytes. Furthermore our study shows for the first time that APM components are also not detectable in normal hepatocytes. Lastly our study shows that HLA class I APM component upregulation is very frequent in HCC. Its association with T cell infiltrate, although not statistically significant, is compatible with the possibility that HCC cells are recognized by CD8+ T cells. If so, HCC should represent an attractive target to apply T cell-based immunotherapy.

